# Fundus autofluorescence and retinal sensitivity in fellow eyes of age-related macular degeneration in Japan

**DOI:** 10.1371/journal.pone.0213161

**Published:** 2019-02-28

**Authors:** Tsutomu Yasukawa, Ryusaburo Mori, Miki Sawa, Ari Shinojima, Chikako Hara, Tetsuju Sekiryu, Yuji Oshima, Masaaki Saito, Yukinori Sugano, Aki Kato, Masayuki Ashikari, Yoshio Hirano, Hitomi Asato, Mayumi Nakamura, Kiyoshi Matsuno, Noriyuki Kuno, Erika Kimura, Takeshi Nishiyama, Mitsuko Yuzawa, Tatsuro Ishibashi, Yuichiro Ogura, Tomohiro Iida, Fumi Gomi

**Affiliations:** 1 Department of Ophthalmology and Visual Science, Nagoya City University Graduate School of Medical Sciences, Nagoya, Japan; 2 Division of Ophthalmology, Department of Visual Sciences, Nihon University School of Medicine, Tokyo, Japan; 3 Department of Ophthalmology, Osaka University Graduate School of Medicine, Osaka, Japan; 4 Department of Ophthalmology, Fukushima Medical University School of Medicine, Fukushima, Japan; 5 Department of Ophthalmology, Graduate School of Medical Sciences, Kyushu University, Fukuoka, Japan; 6 Department of Ophthalmology, Graduate School of Medicine and Faculty of Medicine, Akita University, Akita, Japan; 7 Santen Pharmaceutical Co., Ltd., Ikoma, Japan; 8 Japan Innovative Therapeutics, Inc., Nagoya, Japan; 9 Department of Public Health, Aichi Medical University School of Medicine, Nagakute, Japan; 10 Department of Ophthalmology, Tokyo Women’s Medical University, Tokyo, Japan; 11 Department of Ophthalmology, Hyogo College of Medicine, Nishinomiya, Japan; University of Alabama at Birmingham School of Medicine, UNITED STATES

## Abstract

**Purpose:**

Abnormal fundus autofluorescence (FAF) potentially precedes onset of late age-related macular degeneration (AMD) in Caucasian patients. Many differences exist between Asian and Caucasian patients regarding AMD types and severity, gender, and genetic backgrounds. We investigated the characteristics of abnormal FAF and retinal sensitivity in the fellow eyes of Japanese patients with unilateral neovascular AMD.

**Methods:**

Sixty-six patients with unilateral neovascular AMD and abnormal FAF in the fellow eye were enrolled in this multicenter, prospective, observational study. The best-corrected visual acuity, fundus photographs, FAF images, and retinal sensitivity on microperimetry were measured periodically for 12 months. The FAF images were classified into eight patterns based on the International Fundus Autofluorescence Classification Group. The points measured by microperimetry were superimposed onto the FAF images and fundus photographs and classified as “within,” “close,” and “distant,” based on the distance from the abnormal FAF and other findings. The relationship between the location of the baseline abnormal FAF and retinal sensitivity was investigated.

**Results:**

In Japanese patients, patchy (33.3%) and focally increased (30.3%) patterns predominated in the abnormal FAF. Intermediate-to-large drusen was associated predominantly with hyperfluorescence and hypofluorescence. Neovascular AMD developed within 1 year in six (9.1%) eyes, the mean baseline retinal sensitivity of which was 12.8 ± 4.7 dB, significantly (p<0.002) lower than the other eyes. In 44 of the other 60 eyes, microperimetry was measurable at baseline and month 12 and the mean retinal sensitivity improved significantly from 13.5 ± 4.4 to 13.9 ± 4.8 dB (p<0.001), possibly associated with lifestyle changes (e.g., smoking cessation, antioxidant and zinc supplementation). The mean retinal sensitivities of points within and close to the abnormal FAF were 9.9 and 11.7 dB, respectively, which were significantly lower than the 14.0 dB of the points distant from the abnormal FAF.

**Conclusion:**

In Japanese patients, patchy and focally increased patterns predominated in the abnormal FAF. The retinal sensitivity was lower close to/within the abnormal FAF. FAF and microperimetry are useful to assess macular function before development of neovascular AMD or geographic atrophy.

## Introduction

Age-related macular degeneration (AMD) is a progressive retinal degenerative disease and a common cause of blindness and visual disability in elderly patients in developed countries [1]. There are two advanced types of AMD: non-neovascular and neovascular. The former is characterized by retinal pigment epithelium (RPE) death and underlying choriocapillaris loss leading to geographic atrophy (GA) with or without quiescent choroidal neovascularization (CNV), and the latter by exudation from CNV. In late AMD, both types induce cone photoreceptor death, resulting in severe central visual loss. Photodynamic therapy (PDT) and anti-vascular endothelial growth factor (VEGF) therapy are currently the standard treatments for neovascular AMD [2–7]. Anti-VEGF therapy effectively sustains or improves the visual acuity (VA) in most patients with neovascular AMD. However, repeated injections are required in many cases and are associated with high cost and possible ocular and systemic complications [8–12]. Because no satisfactory treatment exists for GA, it is important to prevent progression to late AMD. Understanding the clinical features of the earlier stages of AMD and the fellow eyes of those with unilateral neovascular AMD might be essential to develop a new prophylaxis for AMD.

Aging is a definite risk factor for AMD, suggesting the role of long-term accumulation of ocular aging changes in the pathogenesis [7,13]. The clinical features of AMD-associated aging changes are the presence of medium (>63 μm and <125 μm) and large (>125 μm) drusen and definite hyperpigmentary or hypopigmentary RPE abnormalities, which are the hallmarks of early-to-intermediate AMD [7,14]. Before progression to late AMD, dysfunction of the rod photoreceptors has been reported and associated with impaired dark adaptation [15–17]. Therefore, it is important to investigate retinal functions and their relationship to fundus findings in the early stages of AMD.

Drusen are extracellular deposits of membranous debris and lipids between the RPE basal lamina and the inner collagenous layer of Bruch’s membrane [18]. More recently, subretinal drusenoid deposits in the subretinal space are considered to be specifically and independently associated with late AMD, especially GA and retinal angiomatous proliferation (RAP) [19]. In addition, abnormal distributions of fundus autofluorescence (FAF) are considered valuable findings in the progression of AMD. Research on FAF has been advancing because of the development of fluorescein angiography devices, which enable noninvasive observation of FAF in the clinic. In general, while background FAF is derived from accumulation of lipofuscin in the RPE cells, abnormal FAF with localized distribution of hyperfluorescence and/or hypofluorescence is sometimes observed and considered relevant to retinal functions and useful for monitoring AMD progression [20,21]. Many patterns of abnormal FAF have been reported and classified internationally. The relationship of abnormal FAF to macular function and progression of AMD also has been investigated in patients of European descent [21–23]. However, there are many differences between Asian and Caucasian patients in the ratio of atrophic and neovascular AMD, the ratio of typical CNV and polypoidal choroidal vasculopathy (PCV), gender, genetic backgrounds, and the responses to PDT and other treatments [24–32]. Therefore, the distribution of patterns of abnormal FAF and the relationship between abnormal FAF and macular function remain to be elucidated in Japanese populations. Based on this, we designed a multicenter, prospective, noninterventional, observational study to investigate the characteristics of the fellow eyes of those with neovascular AMD in a Japanese population and the relationship among abnormal FAF, color fundus photography findings, and temporal changes in retinal functions involving the VA and macular retinal sensitivity measured by microperimetry.

## Materials and methods

The Japanese Fundus Autofluorescence and Microperimetry in Early Age-Related Maculopathy (JFAM) Study was conducted between December 2006 and July 2009 at five university hospitals in Japan. The study adhered to the tenets of the Declaration of Helsinki. The institutional ethics committees at Nagoya City University Graduate School of Medical Sciences, Nihon University School of Medicine, Osaka University Graduate School of Medicine, Fukushima Medical University School of Medicine, and Kyushu University Graduate School of Medical Sciences reviewed and approved the study protocol. Patients provided written informed consent before entry into the study.

### Study population

Eligible patients were Japanese and 50 years of age or older with unilateral late AMD. The fellow eyes of those with neovascular AMD were selected for the study. The inclusion criterion for the study eye was hyperfluorescence or hypofluorescence on FAF images measured by the Heidelberg Retina Angiogram Digital Angiography System (HRA) or HRA2 (Heidelberg Engineering, Heidelberg, Germany). The major exclusion criteria for the study eye were any exudative findings including CNV, hemorrhage, serous RPE detachment, serous retinal detachment, and hard exudates; GA involving the fovea; diabetic retinopathy; uveitis; myopia of 8 diopters or more; retinal vein occlusion; hazy media interfering with fundus examinations; and a history of laser photocoagulation.

After eligibility was determined and written informed consent was obtained, the participants were monitored for 1 year. Each participant underwent measurement of the best-corrected VA (BCVA), binocular funduscopy, and color fundus photography at baseline and months 3, 6, 9, and 12 and FAF imaging and fundus microperimetry at baseline and months 6 and 12.

### Fundus autofluorescence

FAF imaging was performed using HRA or HRA2. To amplify the autofluorescence signal, nine consecutive single images were selected for automatic alignment, and the averaged image was evaluated. In cases with small pupils or media opacity, the examiner changed the setting to more than 9 images to achieve better quality images.

### Microperimetry

Macular sensitivity was measured by a microperimetry device (MP-1, Nidek Technologies, Padova, Italy) using Nidek Advanced Vision Information System software (Nidek Technologies. The following parameters were used in the current study: a customized radial grid of 45 stimuli covering the central 12 degrees, stimuli size Goldman III with a presentation time of 200 milliseconds, and 0- to 20-dB stimulus light intensity of 4-asb white light as the background. A 4–2 stimuli strategy was used, i.e., when a patient did not respond to the first stimulus of 10 dB, the stimulus was increased in 4-dB increments until the patient responded and then was decreased by 2 dB as a final check and vice versa. Chen et al. reported that a point-wise change of 3 dB or greater exceeded the 95% confidence interval of coefficients of repeatability [33]. A 4-dB or greater decrease in the retinal sensitivity was considered clinically significant.

### Image analysis

**Fig 1:**
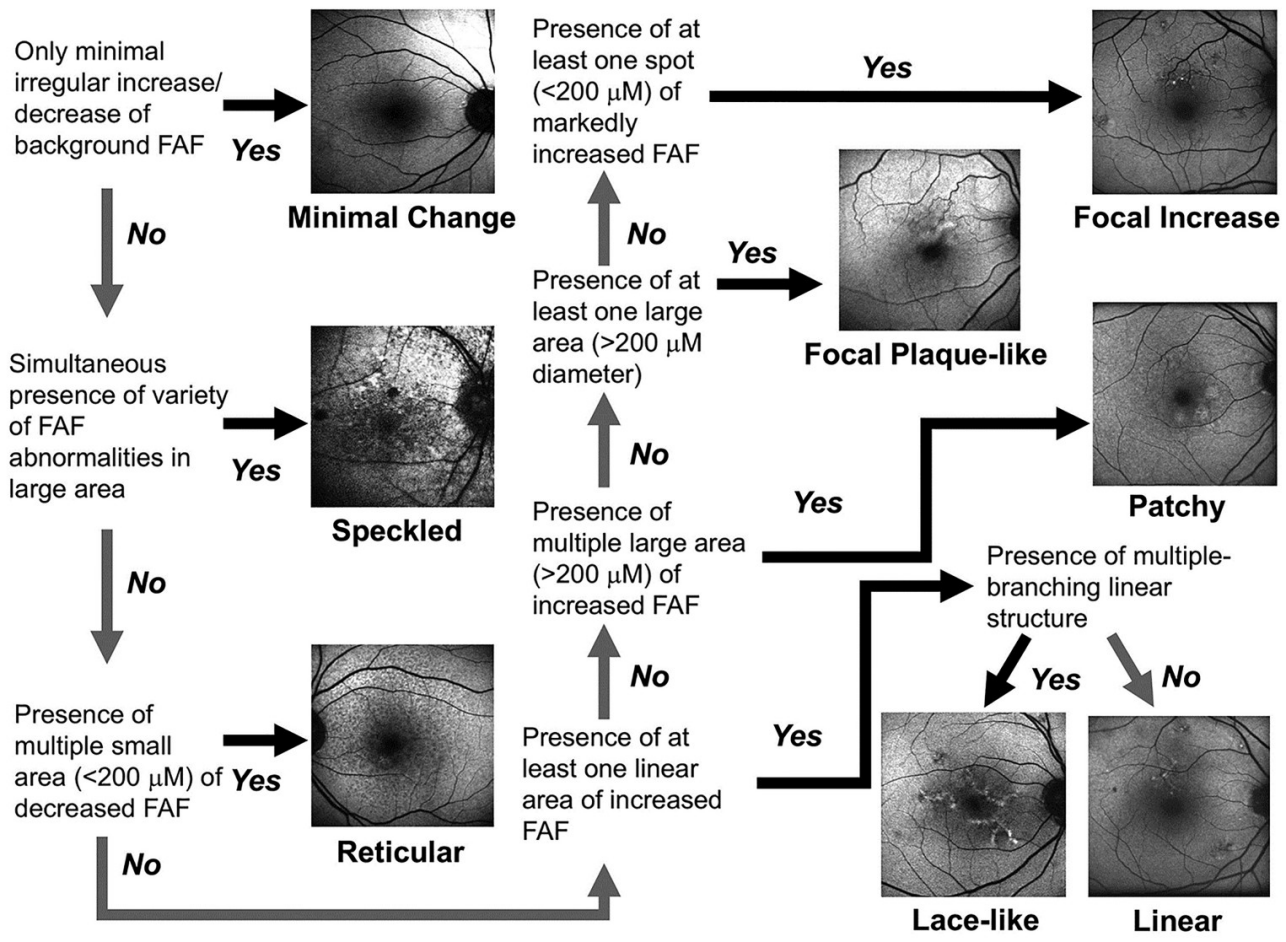
Flow chart for classifying abnormal FAF.

FAF patterns were determined according to the classification of the International Fundus Autofluorescence Classification Group (IFAG) [20,21]. Briefly, three independent observers evaluated all images and classified the findings into eight patterns: minimal change, focal increase, focal plaque-like, patchy, linear, lace-like, reticular, and speckled. The JFAM study group created a flow chart to facilitate pattern classification that was based on the IFAG definition (Fig 1).

We used a flow chart to evaluate the FAF patterns. When an observer’s decision differed from the others, the decision of the two observers who agreed was recorded. When the opinions of the three observers differed, a fourth observer’s decision was considered.

Hyperfluorescent and hypofluorescent areas were demarcated manually. These images then were overlaid on fundus photographs to assess colocalization of hyperfluorescent and hypofluorescent spots with findings on color funduscopy. The baseline retinal sensitivity maps on microperimetry also were overlaid on the baseline FAF images using dedicated MP-1 software. Every retinal point tested with microperimetry was classified into three groups, i.e., “within,” “close,” and “distant,” based on the distance from the abnormal FAF detected on the FAF images and any findings on color fundus photography. The group described as within included the retinal points that overlapped any abnormal findings; the group described as close included the retinal points within 1 degree of the border of any abnormal findings; the group described as distant included points 1 degree or more from any abnormal findings. The mean retinal sensitivity was calculated for each group at each time point. The average change from baseline at each time point also was assessed.

### Statistical analysis

To investigate the association between the retinal sensitivity and distance from the abnormal FAF, hierarchical linear mixed-effect models were used with the distance, time from baseline (months), and angle (degrees) as fixed effects. Differences among patients, eyes, and test point locations were considered successively nested random effects, *i*.*e*., test point locations nested within eyes within patients. We used two models, the first of which included a random intercept for each patient, each eye nested within each patient, and each test point location nested within each eye within each patient (model 1). The second model added the corresponding random slopes to the first model (model 2). Akaike’s information criterion (AIC) and Bayesian information criterion (BIC) were used to compare the statistical models used. Both criteria are parsimony-adjusted indices useful for examining the fit of competing models. These criteria are based on the log likelihood value of a given model and impose a penalty on over-parameterized models. With these statistics, the preferred model is associated with lower relative values [34].

Analysis was performed with the nlme package in R [Jose Pinheiro DB, DebRoy Saikat, Sarkar Deepayan, the R Core team (2018). nlme: Linear and Nonlinear Mixed Effects Models]. All analyses, except as otherwise noted, were performed using the SAS version 9.2 statistical package (SAS Institute Inc., Cary, NC, USA). Analysis of variance (ANOVA) with post-hoc tests, unpaired t-test, or Fisher’s exact test was used for comparisons among groups. The paired t-test was used to compare values with baseline. P<0.05 was considered significant.

## Results

### Patient entry and the onset of AMD

Sixty-nine fellow eyes with abnormal FAF in 69 patients with unilateral neovascular AMD were enrolled; three subjects withdrew from the study because they met an exclusion criterion after study entry. The remaining 66 subjects were followed for 12 months. Table 1 shows the baseline patient characteristics. During the 12-month follow-up, six (9.1%) eyes developed exudative changes related to neovascular AMD and the patients left the study. Nine patients could not be followed beyond 12 months.

**Table 1:** Baseline profiles of 66 study patients.

Age (mean ± SD, years)	73.7±7.5
Gender	
Male	48 (72.7%)
Female	18 (27.3%)
Diagnosis of study eye—no. (%)	
Medium-to-large drusen and/or pigmentary abnormalities	65 (98.5%)
Extrafoveal GA	1 (1.5%)
Diagnosis of fellow eye—no. (%)	
Typical neovascular AMD	46 (69.7%)
PCV	19 (28.8%)
RAP	1 (1.5%)

SD, standard deviation.

### Visual acuity and retinal sensitivity

**Fig 2:**
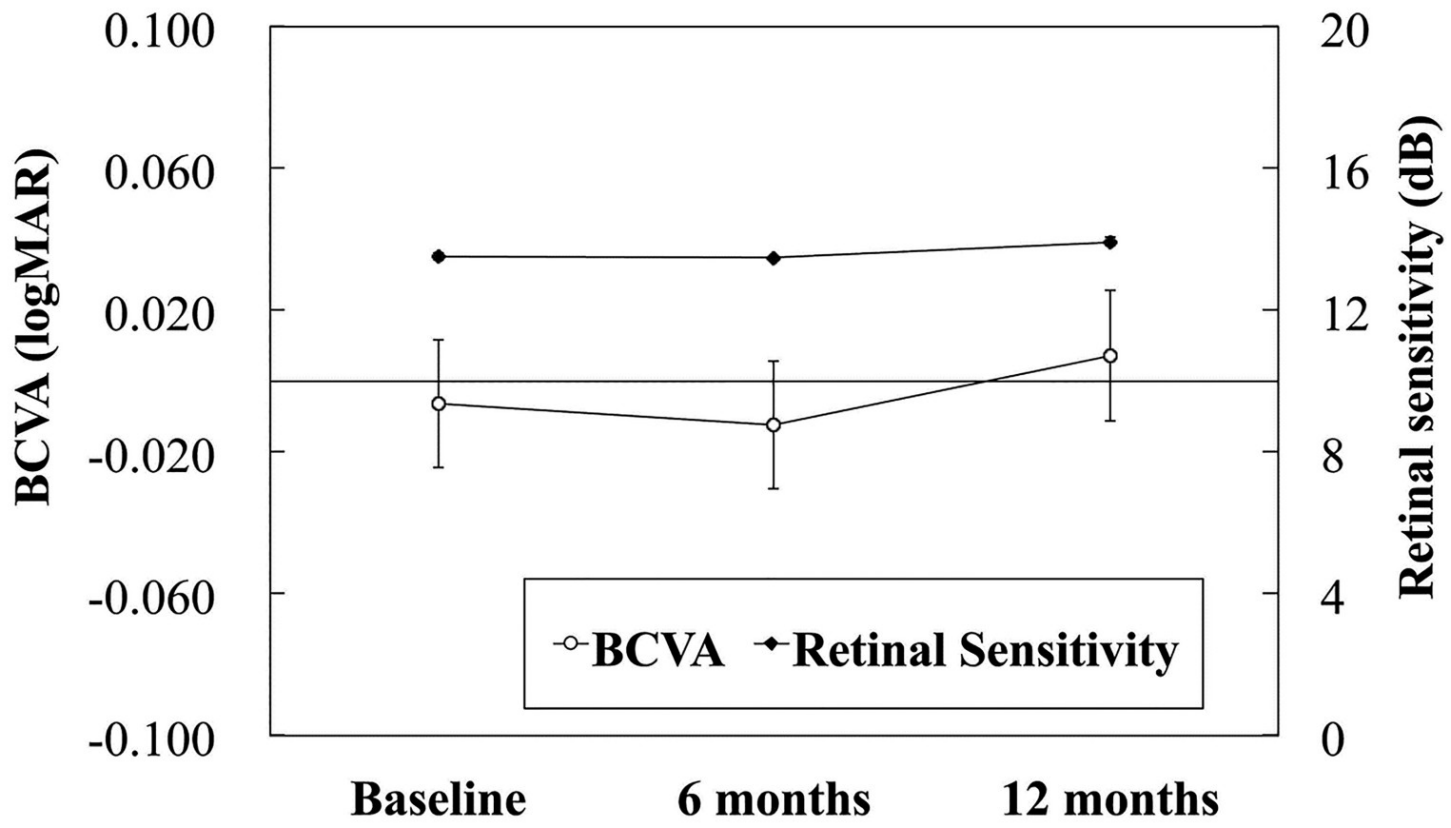
Changes in the mean BCVA logMAR and mean retinal sensitivity during the follow-up period. The data are expressed as the mean ± standard error of the mean. *P<0.01 compared with baseline (paired t-test).

The mean baseline logarithm of the minimum angle of resolution (logMAR) BCVA in 66 eyes was -0.011 ± 0.128. The mean BCVA of 51 eyes with 12-months follow-up decreased from -0.006 ± 0.131 at baseline to 0.007 ± 0.134 at month 12, a change that did not reach significance (P = 0.149, paired t-test) (Fig 2).

The retinal sensitivity within 6 degrees of the fovea was measurable in 63 eyes. An average baseline retinal sensitivity was 13.5 ± 4.5 dB. The mean baseline retinal sensitivity of the six eyes in which AMD developed was 12.8 ± 4.7 dB, significantly (P<0.002, unpaired t-test) lower than the other 57 eyes with 13.7 ± 4.3 dB.

The mean retinal sensitivity of the total 1,976 points in 44 eyes with 12-months follow-up improved significantly (P<0.001, paired t-test) from 13.5 ± 4.4 to 13.9 ± 4.8 dB in month 12 (Fig 2).

### Fundus findings

Color fundus photographs of sufficient quality were available at baseline in 62 eyes at which time hard drusen were detected in 19 (30.6%) eyes, medium-to-large drusen in 53 (85.5%) eyes, hyper-pigmentation in 26 (41.9%) eyes, and hypo-pigmentation or atrophy in 17 (27.4%) eyes. Sixty-one (98.4%) eyes had medium-to-large drusen, retinal pigment alterations, or both. Confluent drusen were observed in 25 of 53 eyes with medium-to-large drusen. Colocalization of hyperfluorescent and hypofluorescent spots with each finding on color funduscopy was assessed. Soft drusen were related to 43% of the hyperfluorescent spots and 39% of the hypofluorescent spots, while most other hyperfluorescent and hypofluorescent spots had no specific findings on color funduscopy.

### Patterns of abnormal FAF

FAF images of sufficient quality were obtained at baseline in 66 eyes and classified into eight patterns according to the IFAG classification (Fig 1, Table 2). No eye with a normal pattern was enrolled in this study. The patchy pattern and the focally increased pattern were predominant.

**Table 2:** Proportion and retinal sensitivity of abnormal FAF patterns. SD, standard deviation.

Patterns of abnormal FAF	n (%)	Retinal sensitivity (dB) (mean± SD)	% of points with 11 dB or better
Minimal change	3 (4.5)	14.5 +/- 2.3	97
Focally increased	20 (30.3)	14.6 +/- 4.1	84
Linear	5 (7.6)	14.6 +/- 3.7	87
Focal plaque-like	4 (6.1)	14.4 +/- 3.4	83
Reticular	4 (6.1)	13.2 +/- 4.5	72
Patchy	22 (33.3)	13.1 +/- 4.8	72
Lace-like	6 (9.1)	11.8 +/- 4.0	63
Speckled	2 (3.0)	9.8 +/- 3.2	40
Total	66 (100)	13.5 +/- 4.5	77

### Abnormal FAF and retinal sensitivity

The mean retinal sensitivity in eyes with each abnormal FAF pattern differed among the abnormal FAF patterns (P<0.01, ANOVA) (Table 2). The speckled pattern had the lowest mean retinal sensitivity of 10 dB or lower at 60% of the measurement points, followed by the lace-like, patchy, and reticular patterns. In the reticular pattern, the retinal sensitivity was relatively preserved, although the fundus was diffusely affected.

**Fig 3:**
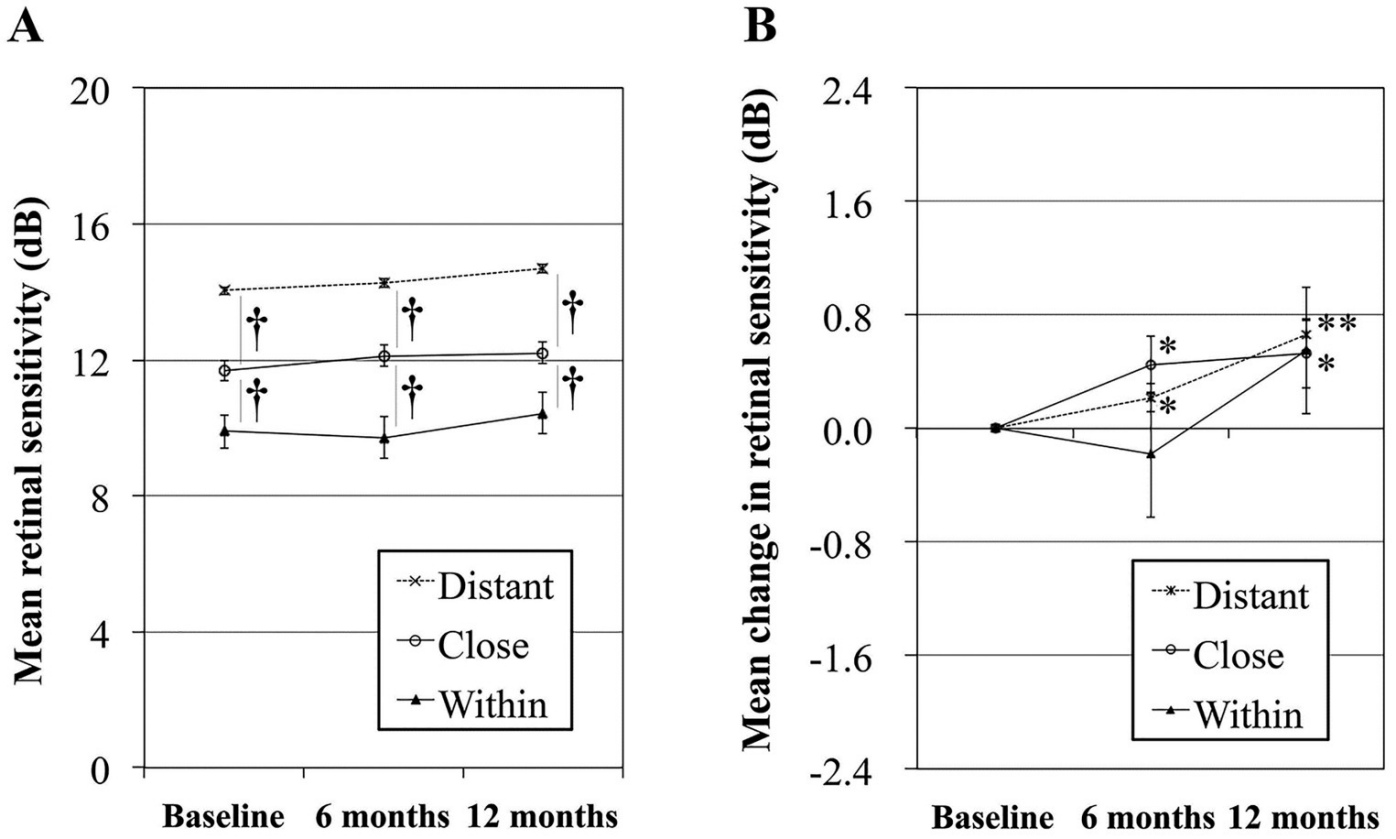
The relationship between retinal sensitivity and the distance from the abnormal FAF. (A) The temporal changes in the mean retinal sensitivity in three groups. (†P<0.01 vs. close at each time point, ANOVA). (B) The changes in the mean retinal sensitivity from baseline in the three groups. The data are expressed as the mean ± standard error of the mean. (*P<0.05 and **P<0.01 as compared with baseline, paired t-test).

In total, 1,550 retinal points in 36 eyes with a 12-month follow-up that included the retinal sensitivity measurement and baseline FAF images of sufficient quality were classified into three groups: within, close, and distant based on the distance from the abnormal FAF. Fig 3 shows the mean retinal sensitivity at each time point.

The mean baseline retinal sensitivities of the close and within groups were 11.7 and 9.9 dB, respectively, which were significantly (P<0.01, ANOVA) lower than the 14.0 dB in the distant group. These significant differences among the three groups were sustained for 12 months. The mean retinal sensitivity in each group tended to increase in months 6 and 12 (P<0.05 for the close and distant groups, paired t-test). To investigate the association between the retinal sensitivity and the distance from the abnormal FAF, hierarchical linear mixed-effect models with the distance, time from baseline (months), and test point locations (degrees between the fovea and test points) as fixed effects were used. In a comparison of the two models, model 2 provided a better fit than model 1, with an AIC and BIC of 24891.73 and 24950.12 in model 1, and 24608.28 and 24705.60 in model 2, respectively. Thus, we examined the association between the retinal sensitivity and the distance from the abnormal FAF based on model 2 and found the retinal sensitivity was associated significantly with the distance from the abnormal FAF (Table 3).

**Table 3:** Results of the random intercept and slope model.

Parameter		Estimate	SE	*P* value
Distance	Within	Reference		
	Close	1.18	0.36	0.001
	Distant	2.65	0.33	<0.001
Time		0.05	0.02	0.031
Test point location (degrees)		0.29	0.04	<0.001

SE, standard error of the mean.

**Fig 4:**
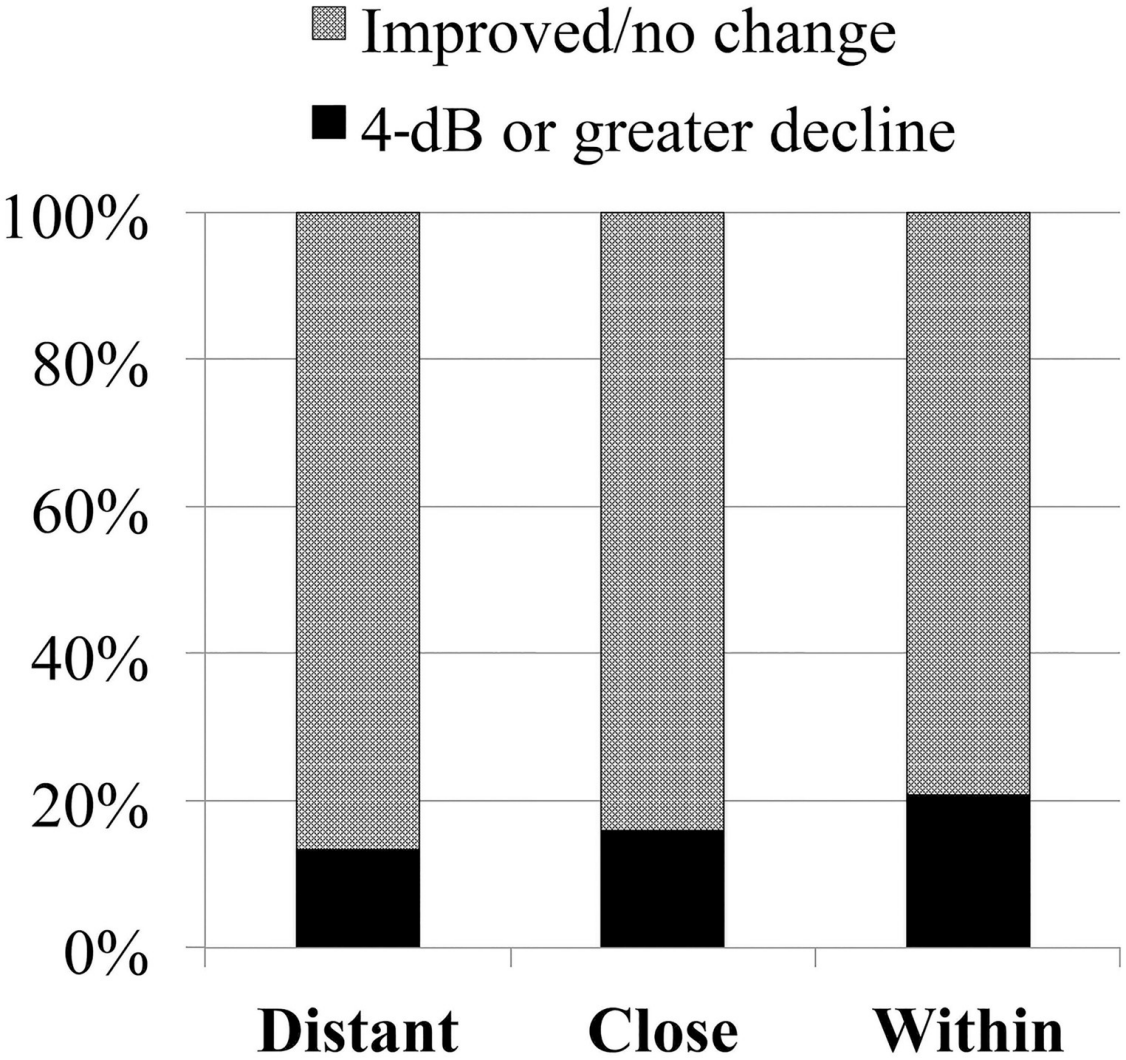
The proportion of points with a 4-dB or greater decline in the groups in which the distances to the abnormal FAF differed (P = 0.113, Fisher’s exact test).

When we counted the number of retinal points with a 4-dB or greater decline in retinal sensitivity at 12 months, 13.3% of the distant points, 15.9% of the close points, and 20.7% of the within points had decreased retinal sensitivity. The differences among the groups were not significant (P = 0.113, Fisher’s exact test) (Fig 4).

## Discussion

It is generally difficult to assess progression of clinical findings such as drusen and pigment changes in eyes with early-to-intermediate AMD, while the incidence of AMD definitely increases with age [7,13,14]. The Age-Related Eye Disease Study (AREDS) Research Group created the AMD categories based on abnormal findings seen on color fundus photographs. Category 4, the most severe condition, is characterized by unilateral CNV or GA and in the fellow eye the VA is 20/32 or better [35]. Another study reported that the probability of progression to bilateral advanced AMD was 24.4% at 2 years in eyes with category 4, which was much higher than other categories [36]. However, in Japanese populations, the prevalence of late AMD is slightly lower than in Caucasians [7,13,24–28,35,36]. Moreover, PCV is predominant and unilateral in many cases, while the prevalence rates of RAP and GA, which often affect both eyes, are much lower [28–32]. Because an aim of the current study was to identify eyes with a potential for transition of unilateral neovascular AMD to bilateral neovascular AMD, we enrolled patients without neovascular AMD but abnormal FAF in one eye and neovascular AMD in the other eye. In the current study, six (9.1%) eyes progressed to neovascular AMD during the 12-month follow-up, which might be comparable to the results of the AREDS study. Five of the six eyes were evaluated by fundus photography. All five eyes had soft drusen and four eyes had hyperpigmentation (Table 4).

**Table 4:** Characteristics of six eyes with progression to neovascular AMD.

Case	Age	Month with withdrawal	Mean retinal sensitivity		Abnormal FAF pattern	Hard drusen	Soft drusen	Confluent drusen	Hyper-Pigmentation	Hypo-Pigmentation/atrophy
			baseline	6 M						
1	77	12	11.0	16.6	Linear	-	+	-	-	-
2	70	6	13.3	-	Patchy	+	+	+	+	-
3	75	3	19.7	-	Focal increase	NA				
4	83	12	11.5	8.8	Focal increase	-	+	-	+	+
5	81	12	8.0	9.8	Patchy	-	+	-	+	-
6	60	9	12.4	14.6	Lace-like	+	+	-	+	-

M, months; NA, not analyzed.

In the current study, eyes with abnormal FAF were enrolled. Of them, the prevalence rates of soft drusen and pigmentation were 85.5% and 41.9%, respectively. A previous study reported only a 0.6% incidence of neovascular AMD in the fellow eye of Japanese patients with unilateral AMD [37]. Therefore, abnormal FAF, soft drusen, and pigmentation might indicate a high risk of progression to neovascular AMD or GA.

The Autofluorescence Imaging in Age-related Macular Degeneration (FAM) Study investigated the autofluorescence patterns in 125 eyes with multiple soft drusen in German populations, and the most frequently observed in descending order were the patchy, reticular, focally increased, and minimal change patterns. That study also reported that the areas with a reticular pattern of autofluorescence usually corresponded to areas covered with reticular pseudodrusen in color fundus photographs [22]. In the current study, the most frequently observed pattern was the patchy pattern, as in the FAM study. In contrast, the reticular pattern was seen much less frequently. Although these two studies cannot be compared directly because of differences in eligibility criteria, the difference in the incidence of the reticular pattern between the two studies might indicate differences in environmental factors and/or genetic predispositions associated with AMD between Caucasian and Japanese populations. Reticular pseudodrusen was reported to be associated with GA [23]; the low incidence of atrophic AMD in Japanese populations might account for this [28,38]. In six eyes with exudative AMD, a focally increased pattern was seen in two eyes, a patchy pattern in two eyes, and linear and lace-like patterns in one eye each. Previous reports have demonstrated that the patchy pattern was associated most frequently with progression of CNV [39,40].

**Fig 5:**
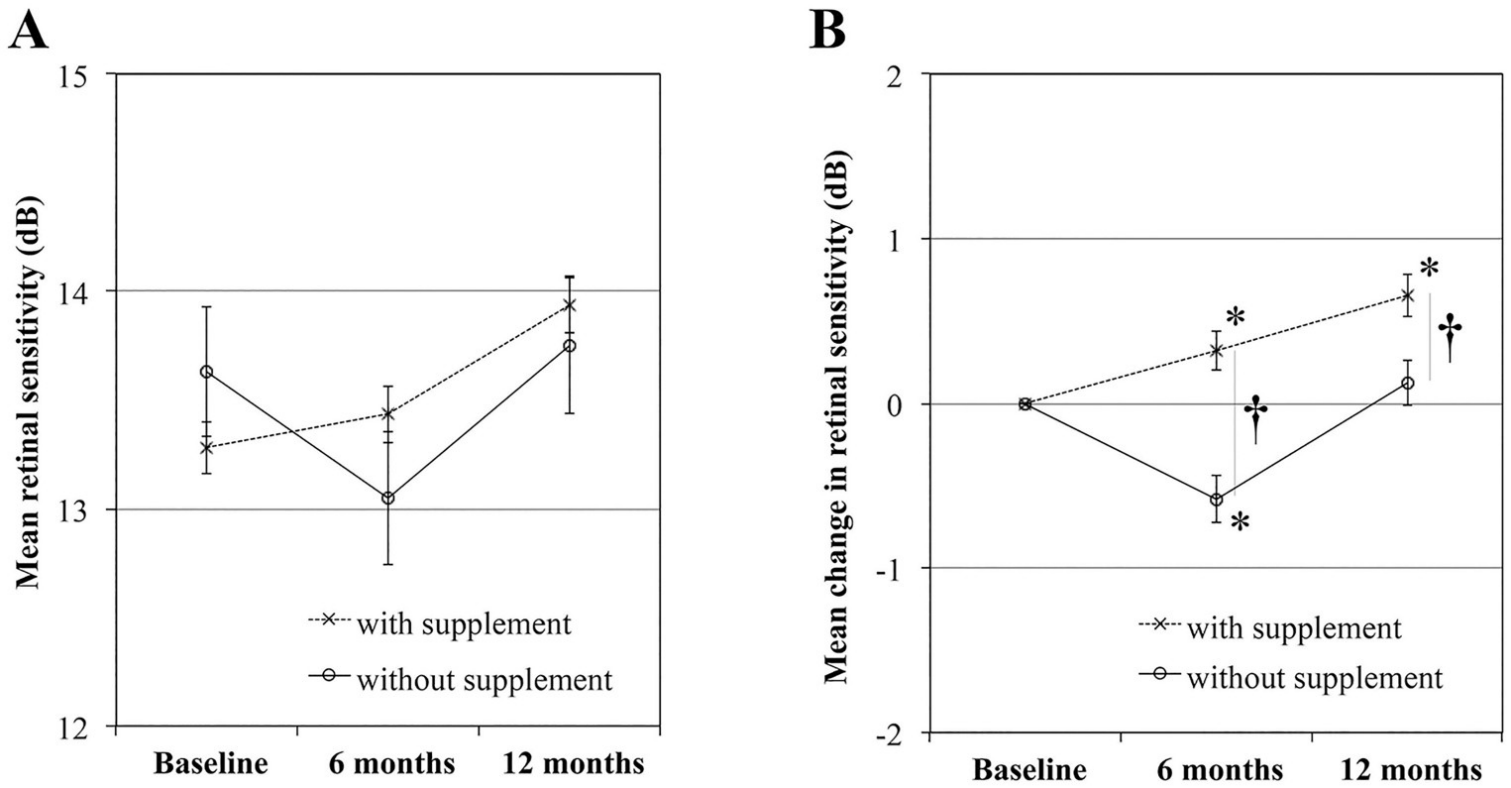
The impact of supplementation on the mean retinal sensitivity. (A) The temporal changes in the mean retinal sensitivity in groups with and without supplementation. (B) The changes in the mean retinal sensitivity from baseline. (†P<0.01, ANOVA; *P<0.01 compared with baseline, paired t-test).

In the current study, the mean BCVA did not change significantly, while the average retinal sensitivity within 6 degrees of the fovea improved in month 12 (Fig 3). Independent of the distance from the abnormal FAF, the retinal sensitivity tended to improve by 12 months (Fig 3). Although the comparison of the large number of points measured might determine that minute medically irrelevant differences were statistically significant, it is noteworthy that the group with supplementation had a significant improvement in retinal sensitivity (Fig 5). Among 44 eyes with complete retinal sensitivity data, six eyes had no information regarding supplementation. Of the other 38 eyes, 20 eyes that received supplementation of antioxidants, lutein, or both according to the AREDS formulation [34] showed a significant increase in the mean retinal sensitivity; 18 eyes that did not receive supplementation had no increase in the mean retinal sensitivity (Fig 5). The differences in the baseline FAF patterns were unlikely to affect temporal changes in the VA or retinal sensitivity during the observation period (data not shown). Sasamoto et al. reported that daily intake of 6 mg of lutein improved the retinal sensitivity even in healthy eyes [41]. Xanthophyll is abundant only at the central 4-degree circle, while only 13 of 45 measurement points on microperimetry are involved in that area. Nevertheless, xanthophyll may be effective not only for blocking against blue-light hazards but also for antioxidation and supplements include other antioxidants. However, the patients were younger and the smoking status was improved in the group with supplementation compared with those without supplementation (Table 5). We speculated that the improved retinal sensitivity in this study might have resulted at least partly from antioxidant supplementation and smoking cessation. However, because patients with or without supplementation were not randomized in the current study, further study is needed to elucidate the prophylactic and/or healing effects of antioxidant supplementation on the retinal sensitivity.

**Table 5:** Characteristics of patients with or without supplementation.

		Antioxidant supplementation		P value
		+	-	
Age		70.2±6.8	75.6±6.9	0.0216
Sex	Men	13	12	1.0000
	Women	7	6	
Smoking	Current	1	5	0.0304
	Ever	8	8	
	Never	11	3	
	Unknown	0	2	

Retinal points close to or within the abnormal FAF already had decreased retinal sensitivity at baseline compared with those distant from the abnormal FAF (Fig 3), a finding consistent with a previous report [21]. Wu et al. reported that reduced retinal sensitivity was associated with the RPE-drusen complex layer thickness and the number of hyperreflective foci in the early stages of AMD [42]. Because the patchy pattern often corresponds to large drusen and drusenoid pigment epithelial detachment, this report may be consistent with our results. The mean baseline retinal sensitivity in the six eyes that progressed to neovascular AMD was already lower than that in eyes without progression to neovascular AMD for 1 year. Thus, measurement of the retinal sensitivity in eyes with early-to-intermediate AMD and eyes with a fellow eye with late AMD might be useful to assess retinal function and the risk of progression to neovascular AMD or GA.

Histologic conditions reflecting each pattern of abnormal FAF remain to be elucidated. Recent histologic investigations have shown that some local hyperautofluorescent spots are derived from RPE cells that sloughed into the subretinal space, nonnucleated RPE fragments shed into basal laminar deposits, or double-layered RPE cells, while some local hypoautofluorescence spots reflect disorganized RPE cells devoid of intracellular granules or lack RPE cells [43–45]. These RPE disorders associated with hyperfluorescent and hypofluorescence may be related to rod dysfunction and decreased retinal sensitivity in the early stages of AMD. Nevertheless, linear, lace-like, and focal plaque-like patterns cannot be explained by the distribution or aggregation of disordered individual RPE cells. Reticular and specked patterns that widely cover the retina beyond the macular area also are not explainable by the behavior of individual RPE cells. Quantitative FAF measurement showed that the intensity of lipofuscin-related FAF decreased in early, intermediate, and late AMD [46]. The intensity of FAF in eyes with subretinal drusenoid deposits, which often shows a reticular pattern, decreases [47]. In the current study, the mean retinal sensitivity in eyes with a speckled pattern was much worse than in eyes with a reticular pattern. We speculated that the speckled pattern might arise from diffusely disordered RPE cells and the reticular pattern might reflect not only a decrease in the lipofuscin granules in impaired RPE cells but also blockage of excitation and/or emission light.

The current study had some limitations. Because of the small number of eyes enrolled and ophthalmologists who performed subjective evaluations detected the abnormal FAF for enrollment, the proportion of the abnormal FAF patterns in this Japanese population may be inaccurate. Although MP-1 was used for microperimetry, recently introduced more advanced ophthalmic devices can performed both photopic and scotopic microperimetry. Curcio and colleagues demonstrated that rod is damaged more earlier than cone photoreceptors [15–17]. In areas with subretinal drusenoid deposits, only scotopic microperimetry showed decreased retinal sensitivity [47,48]. Thus, scotopic microperimetry may be more sensitive for detecting early functional impairment. In addition, optical coherence tomography (OCT) and OCT angiography enable structural analysis and assessment of hemodynamics in areas with abnormal FAF [49].

## Conclusions

The patchy pattern of abnormal FAF predominates in Japanese populations as in Caucasian populations. In contrast, the reticular pattern was seen much less frequently in Japanese patients, which is consistent with the low rate of atrophic AMD in Japan. Abnormal FAF might be a valuable finding as are drusen and pigment changes in eyes with early-to-intermediate AMD and eyes with a fellow eye with late AMD. Microperimetry might be useful to assess macular function in these eyes, predict the progression to advanced stages of AMD, and assess the efficacy of possible prophylactic treatments in future studies.
